# Strengthening initial teacher education programs to deliver confident, effective, classroom-ready graduates: a feasibility study

**DOI:** 10.1186/s40814-025-01713-6

**Published:** 2025-11-03

**Authors:** Nicholas Riley, Angus A. Leahy, Narelle Eather, Simone J. J. M. Verswijveren, Natalie Lander

**Affiliations:** 1 Centre for Active Living and Learning, College of Human and Social Futures, University of Newcastle, Callaghan, Newcastle, NSW Australia; 2 School of Exercise and Nutrition Sciences, Institute for Physical Activity and Nutrition (IPAN), Deakin University, Geelong, Australia

## Abstract

**Background:**

TransformEd is an evidence-based program that targets initial teacher education to equip future teachers with innovative strategies that increase the health, wellbeing and education outcomes of school children. Feasibility, effectiveness and implementation research demonstrate that TransformEd significantly enhances pre-service teachers’ confidence and competence to deliver such strategies. However, to date, the program has only been tested in undergraduate teaching degrees. It is important to gather more evidence for the feasibility and efficacy of the program at all levels of initial teacher education, including at postgraduate level.

**Methods:**

A feasibility study was conducted to examine the feasibility of embedding TransformEd in a Master of Teaching degree at one Australian university. Preliminary efficacy was measured by pre- and post-program surveys completed by Master of Teaching pre-service teachers who received the 12-week TransformEd program. Implementation was measured by adherence checklists completed by lecturers who delivered TransformEd. Post-program focus groups and interviews with senior academics and lecturers, respectively, explored perceptions of all eight domains of feasibility.

**Results:**

Survey results demonstrated that the TransformEd program was positively received by pre-service teachers (n = 21), resulting in improvements in several measures of teaching confidence and competence regarding delivering strategies to improve school children’s health, wellbeing and learning. The TransformEd strategies were well adopted by lecturers and implementation increased over time. Both lecturers (n = 3) and senior academics (n = 2) had positive perceptions of the program, highlighting its feasibility in postgraduate initial teacher education.

**Conclusion:**

This study supports the feasibility and preliminary efficacy of the TransformEd program in postgraduate initial teacher education and provides a foundation for a future controlled trial to rigorously evaluate its impact, with potential population-level benefits for children's health, wellbeing, and educational outcomes in Australia.

**Supplementary Information:**

The online version contains supplementary material available at 10.1186/s40814-025-01713-6.

## Key messages regarding feasibility

• What uncertainties existed regarding the feasibility?

Feasibility, effectiveness and implementation research demonstrate that TransformEd significantly enhances pre-service teachers’ confidence and competence in regard to delivering strategies to improve children’s health, wellbeing and education outcomes. However, to date, the program has only been tested in undergraduate teaching degrees. It is important to gather more evidence for the feasibility and efficacy of the program at all levels of initial teacher education, including at postgraduate level. Therefore, a feasibility study was conducted to examine the suitability and practicality of embedding TransformEd in a Master of Teaching degree at one Australian university.

• What are the key feasibility findings?

Survey results demonstrated that the TransformEd program was positively received by pre-service teachers, resulting in improvements in several measures of teaching confidence and competence in regard to delivering strategies to improve school children’s health, wellbeing and learning. The TransformEd strategies were well adopted by lecturers and implementation increased over time. Both lecturers and senior academics had positive perceptions of the program, highlighting its feasibility in postgraduate initial teacher education.

• What are the implications of the feasibility findings for the design of the main study?

This study provides support for the feasibility and preliminary efficacy of the TransformEd program in the context of postgraduate initial teacher education. The findings have direct implications for the progression of TransformEd from feasibility to a future controlled trial to rigorously evaluate the intervention’s efficacy in postgraduate initial teacher education, which may potentially have a population-level impact on the health, wellbeing and education outcomes of all Australian children.

## Background

Strong and consistent evidence supports the importance of regular physical activity for the physiological and psychological health of children and adolescents [1–3]. Physical activity can improve outcomes relating to physical health, mental health and well-being [1–3]. Emerging evidence also supports the relationship between physical activity and key educational outcomes, such as school engagement, cognition and academic achievement [4, 5]. Consequently, the World Health Organization recommends that children and adolescents participate in at least 60 min of physical activity per day to achieve optimal health and development outcomes [6]. Further, when individuals participate in more physical activity and/or physical activity performed at higher intensities, they experience more potent and additional health benefits [7–10]. However, current data demonstrates that approximately 80% of children and adolescents globally [11] and across Australia [12] are considered insufficiently active.

Schools are regarded as an excellent setting for providing physical activity opportunities for children and adolescents, especially when using a whole-of-school approach [13–15]. The World Health Organization promotes six evidence-based domains for promoting physical activity in schools via a whole-of school approach, including active classrooms and quality physical education [14]. Yet children often spend up to 80% of a school day sitting or in sedentary behaviour [16]. Despite the fact that most countries have legal requirements for providing physical education during the compulsory schooling years, non-compliance and/or lack of regulation is evident in a large proportion of schools [14]. The organisational structures and varied priorities observed in schools create unique challenges for implementing feasible, effective and sustainable physical activity programs [17, 18]. As such, there is a need to explore novel strategies to increase physical activity in school-aged children, beyond physical education, so that the associated health and educational outcomes can be realised.

In New South Wales, Australia, curriculum recommendations require primary school students to spend 25–35% of a school week in English lessons, 20% in mathematics lessons and only 6–10% in personal development, health and physical education lessons [19]. Given that physical education represents the primary context within schools for developing children's movement skills and knowledge to support lifelong physical activity, it is unsurprising that Australian children continue to perform poorly for physical activity participation [12]. Interestingly, and despite the strong emphasis on literacy and numeracy in Australian schools, national and international assessment data indicate that Australian students are scoring below average in reading, science and mathematics as measured by the Programme for International Student Assessment, and these scores have been decreasing over time [20]. Given the link between physical activity and improved educational outcomes, there is a strong rationale for reconsidering the pedagogical approach of English and mathematics programs in Australian primary schools, and reinvigorating lessons with movement-based learning. Recent intervention studies conducted by our research team, amongst others, support this approach, in which combining curricula with meaningful physical activity has shown to facilitate improvements in children’s physical and psychological health and wellbeing, as well as educational outcomes [16, 21–24].

Despite the effectiveness of quality physical activity programs in schools, sustained implementation is often limited due to teacher-reported barriers such as time constraints and a crowded curriculum [25–28]. Integrating meaningful physical activity in academic lessons has the potential to address these barriers [14]. However, few research studies involving physically active academic lessons have been translated into practice or implemented at scale [29], thus producing little evidence of sustainability and/or widespread dissemination [30].

Providing adequate training and support during the implementation of research-based initiatives, as well as extending support beyond the funding period through continued professional learning for teachers, has been recommended to effectively implement active lessons in schools long term [31, 32]. However, it has been argued that many professional development or professional learning models are not optimal for sustained changes in pedagogical practice [33] even with organisational and environmental support. A novel approach for maximising widespread adoption and sustainability of school-based physical activity programs may lie in embedding movement-based or physically active learning in initial teacher education programs [34].

TransformEd is an evidence-based, innovative, behavioural, pedagogical and environmental intervention that targets initial teacher education to equip future teachers with innovative strategies to increase physical activity in the classroom to simultaneously generate children’s health and educational outcomes. The program is directly aligned with the Australian Professional Standards for Teachers: Graduate teacher level [35] and, therefore, supports the development of critical teacher capabilities to deliver confident, effective, classroom-ready graduates. Feasibility [34] and implementation [36] studies conducted in an undergraduate teacher education degree at one Australian university indicated that TransformEd significantly enhanced pre-service teachers’ willingness to deliver active pedagogies as well as their perceived teaching confidence and competence. A subsequent hybrid implementation-effectiveness trial investigated the effects of the TransformEd program when embedded in undergraduate teacher education degrees at two Australian universities compared with a third ‘usual practice’ control university [37]. Results indicated favourable intervention effects on teaching competence and confidence amongst pre-service teachers compared with a control group, and qualitative data suggested that the program strengthened the connection between theory and practice (i.e. between pre-service or initial teacher education and actual teaching requirements when in primary schools).

Although TransformEd has demonstrated feasibility, implementation and positive outcomes in relation to pre-service teachers’ perceived teaching competence, this initiative has only been tested in undergraduate teaching degrees (i.e. Bachelor of Education). However, a bachelor degree is only one type of initial teacher education program that can be undertaken by primary school teachers. The percentage of public school teachers who hold a postgraduate degree has increased over the past decade [38, 39]. In Australia, approximately one-third of teachers have a postgraduate degree (e.g. Master of Education) [40]. Consequently, it is important to gather more evidence for the feasibility and efficacy of the TransformEd program at all levels of initial teacher education, including postgraduate.

Therefore, the aim of this feasibility study was to (i) assess the preliminary efficacy of the TransformEd program on Master of Teaching pre-service teachers’ confidence and competence in teaching, and willingness to integrate strategies to improve children’s health, wellbeing and education outcomes, into current and future teaching practice; (ii) investigate lecturers’ implementation of the TransformEd key strategies; and (iii) examine the perceptions of feasibility of embedding the TransformEd program in the Master of Teaching degree, among lecturers and senior academics.

## Methods

### Design

A mixed method feasibility study was conducted to examine embedding the TransformEd program in the Master of Teaching (Primary) degree at the University of Newcastle, Australia. Bowen and colleagues [41] identified eight areas to address in a feasibility study: demand, acceptability, implementation, practicality, adaption, integration, expansion and efficacy (see Table 1). Each of these were investigated in the present study, with a particular focus on preliminary efficacy and implementation.

**Table 1:** Feasibility focus areas (Bowen et al., 2009)

Focus area	Definition	Sample outcomes of interest
Acceptability	The extent the new program is perceived as suitable to the program deliverers	Satisfaction Intent to continue use Perceived appropriateness Fit into organisational culture
Demand	The extent the new program is likely to be used (how much demand is likely to exist)	Fit into organisational culture Perceived positive or negative effects on the organisation Expressed interest or intention to use Perceived demand
Implementation	The extent, likelihood and manner in which the new program can be successfully delivered as intended or planned	Degree of execution Success or failure of execution Amount and type of resources needed Factors affecting implementation ease or difficulty
Practicality	The extent to which the program can be delivered using existing resources, means and circumstances without outside intervention	Efficiency, speed or quality of implementation Positive/negative effects on target participants Ability of participants to carry out intervention activities
Adaptation	The extent the program performs when changes are made for a different context or populations	Degree to which similar outcomes are obtained in new format
Integration	The extent a new program can be integrated into an existing system	Perceived fit with infrastructure Perceived sustainability
Expansion	The extend an already successful intervention can be expanded to provide a new program or service	Fit with organisational goals and culture Positive or negative effects on organisation Disruption due to expansion component
Limited efficacy	The extent the new program shows promise of being successful with the intended population	Intended effects on program variable Maintenance of change from initial change

In alignment with Bowen et al.’s (2009) framework, we assessed efficacy testing as one of several domains of feasibility, with the aim of determining whether the intervention shows initial promise rather than establishing effectiveness. To assess limited or preliminary efficacy, baseline and follow-up measures (i.e. online surveys) were conducted with Master of Teaching pre-service teachers who undertook the 12-week TransformEd program. To investigate program implementation, an online adherence checklist was completed by each lecturer at three time points across the unit (early: week 3, mid: week 6, late: week 9) to assess lecturers’ adherence to the TransformEd key strategies (Table 2). Post-program focus groups were conducted with senior academics in the School of Education and interviews with lecturers who delivered TransformEd to examine their perceptions of all eight areas of feasibility. Participant responses were analysed in relation to the suggested outcomes of interest (Bowen et al., 2009; Table 1) to determine feasibility of embedding the TransformEd program in a Master of Teaching degree, and thus its appropriateness for further research. Where relevant, the CONSORT checklist [43] for pilot trials informed the study design. The study was approved by Deakin University Human Ethics Committee (HAE-17–207) and the Newcastle University Human Ethics Committee (H-2021–0313).

**Table 2:** TransformEd key concepts and lecturer implementation strategies

Key components	Elaboration	Lecturer implementation strategies
Active academic lessons	Active lessons utilise incidental activity or embodied learning to change the delivery of a traditional seated lesson Active lessons help to create a positive environment for learning, and they also provide a platform for excellence in teaching	Modelled active academic teaching strategies in lectures and practical seminars
		Integrated pedagogical theory (e.g. embodied pedagogy) and practice (e.g. skills, strategies, organisational and managerial concepts) to facilitate active academic lessons
		Provided resources to pre-service teachers for active academic lessons
		Provided opportunity for pre-service teachers to practice skills, strategies, organisational and managerial concepts required to teach active academic lessons
		Provide opportunity for self, peer and lecturer feedback on pre-service teachers’ active academic peer teaching
Active breaks from sitting	Short active breaks interrupt prolonged periods of sitting. There are several different types or purposes of active breaks [42]. They can be used to complement lesson content using physical and visual reinforcement, introduce or summarise lesson content, structure the lesson, transition, the lesson, proactively manage the class and create a positive classroom environment [42]	Modelled active beaks in lectures and seminars
		Integrated pedagogical theory and practice (skills, strategies, organisational and managerial concepts) to facilitate active breaks
		Provided active break resources to pre-service teachers
		Provided opportunity for pre-service teachers to practice skills, strategies, organisational and managerial concepts required to break sitting time
		Provided opportunity for self, peer and lecturer feedback on pre-service teachers’ active break peer teaching
Health lesson curriculum content	Class lessons that aim to build skills and increase knowledge about the importance of being active and sitting less	Provided information around the importance of adequate physical activity
		Provided resources for future teaching around the importance of physical activity
		Provided opportunity for pre-service teachers to practice skills, strategies, organisational and managerial concepts required to deliver physical activity related content in peer teaching
		Provided opportunity for self, peer and lecturer feedback around their physical activity related content peer teaching
Active environments/ promoting activity during recess and lunchtime	Signage, equipment, facilities, resources, policy and teacher encouragement/support to promote physical activity at recess and lunchtime	Delivered seminar/lecture focused on playground-based activities that facilitate PA at recess/lunchtime
		Provided resources for playground-based activities
		Provided opportunity for pre-service teachers to practice skills, strategies, organisational and managerial concepts required to facilitate playground-based activities, in peer teaching
		Provided opportunity for self, peer and lecturer feedback around their playground activities peer teaching
Engaging families	Information and home-based activities provided for parents and children to engage with to reinforce the importance of children being active and sitting less	Delivered seminar/lecture on active homework strategies that engage families and educate around the importance of increasing PA and decreasing sitting time at home
		Provided information around the importance of engaging families and the community when addressing physical activity behaviour (e.g. ecological model)
		Provided active homework resources
		Provided opportunity for active homework activities, peer teaching
		Provided opportunity for self, peer and lecturer feedback around their active homework tasks

### Recruitment and consent

All first-year Master of Teaching (Primary) pre-service teachers (*n* = 41) were invited to participate in the study (March, 2022) via direct emails sent to their university email accounts. A plain language statement was provided, and written consent to participate was required prior to completing the survey. Lecturers responsible for the delivery of the Master of Teaching units identified for the TransformEd program (i.e. Mathematics, English and Personal Development Health and Physical Education) were invited to participate in the study (i.e. professional learning, program delivery, adherence checklists and post-program interviews) via email invitation (*n* = 3). Senior academics (i.e. Course directors and Unit Chair) were invited to participate in a post-program online focus group via email invitation (*n* = 2). Senior academics are crucial decision-makers and gatekeepers regarding degree, course and unit design, structure, curriculum, modes of delivery, assessment and policy. As such, an understanding of the views of these key stakeholders not only helps to inform the feasibility of the program but is also integral to the development of a future definitive trial. The senior academics invited into the study included the Primary Education Program Convenor and the Deputy Head of School for Teaching and Learning, neither of whom teach in the Master of Education program. A plain language statement was provided to senior academics and lecturers, and written consent to participate was required.

### Sample size justification

As the primary aim of this feasibility study was to assess trial processes and intervention implementation, the sample size was determined pragmatically, based on logistical considerations (i.e. the first-year Master of Teaching cohort, *n* = 41), available resources (including the number of units and lecturers involved) and the time frame (one semester). Assuming a response rate of 50% (the point of maximum variance), the 95% confidence interval for our sample size (*n* = 41) was approximately ± 15 percentage points (i.e. 35% to 65%), indicating a moderate degree of precision.

### Intervention

There are five key components and associated implementation strategies to the previously developed and effective TransformEd intervention program: active academic lessons; active breaks from sitting; health curriculum content; active environments and promoting physical activity during recess and lunch breaks; and engaging families (Table 2). All strategies aim to simultaneous improve the health and learning outcomes of the primary school students that the pre-service teachers go on to teach. In addition, the concepts and resources in the program are designed to improve critical teacher capabilities that align with the Australian Professional Standards for Teachers: Graduate standards and descriptors (e.g. Standard 1: Know students and how they learn; Standard 2: Know the content and how to teach it; Standard 3: Plan for and implement effective teaching and learning; Standard 4: Create and maintain supportive and safe learning environments) [35]. The intervention was implemented in three phases.

#### Phase 1

The lead researcher worked with key senior academics (invited and consented *n* = 2) in the School of Education at The University of Newcastle to identify relevant TransformEd units in the first year of the Master of Teaching degree, and lecturers responsible for the delivery of the units. The lead researcher provided a two-hour interactive professional learning workshop to senior academics, which outlined the rationale, importance, key components, evolution and effectiveness of the TransformEd program. During the professional learning workshop (via video conference), the lead researcher provided and explained the comprehensive bank of online TransformEd resources, which includes over 50 2-min active breaks, over 100 active academic lessons and ‘frequently asked questions’, ‘how to’ and ‘getting started’ guides. The resources extend across a range of key learning areas including mathematics, science, English, geography and history.

The active breaks and active academic lesson resources cover all primary levels from Foundation to Grade 6 and are all linked to the Australian and New South Wales curricula. Further, each resource identifies the content description that specifies what students will learn, and the achievement standards that describe the depth of understanding, knowledge and skill expected of students at the end of each year level or band [44]. In addition, the TransformEd resources are designed to support the development of primary school student capabilities (e.g. critical and creative thinking, and personal and social capabilities) and provide cross-curriculum opportunities for students to strengthen their literacy and numeracy capabilities [44]. Collectively, the program provided a whole-of-school approach to concurrently improve the health and learning outcomes of all primary school students.

#### Phase 2

Using a train-the-trainer design, the senior academics shared the professional learning and online resources with lecturers, and then worked with them to co-create, adapt and embed the TransformEd key concepts and implementation strategies into the selected units (see Table 2). The aim was to align the TransformEd program with the learning objectives of each unit and scaffold the program concepts to facilitate the development of unit-specific teacher competencies (e.g. the use of active breaks as a proactive classroom management strategy; the use of active lessons to engage students in learning and embody the learning content in mathematics and English, the use of different types of active breaks to plan and structure lessons).

#### Phase 3

Lecturers then delivered various TransformEd key components in their respective units (i.e. mathematics, English and Personal Development Health and Physical Education). In relation to implementation, the lecturers were encouraged to model the key components and concepts in their own teaching, incorporate the concepts into unit learning curriculum, content and assessment and reinforce the content by providing peer-teaching opportunities. The lecturers were provided with a checklist of key concepts and key implementation considerations for their planning and teaching (see Table 2). Lecturers also made the online resources available to the master’s students to use in their peer teaching and teaching placements.

## Procedures

### Data collection

Mixed methods were used during the 12-week feasibility study to investigate the eight identified areas of feasibility: demand, acceptability, implementation, practicality, adaption, integration, expansion and efficacy, with a particular focus on preliminary efficacy and implementation.

### Limited efficacy testing

Efficacy, within the scope of this feasibility study, is considered preliminary and exploratory in nature, consistent with the aims of feasibility research. To evaluate program *efficacy* at the pre-service teacher level, baseline and follow-up self-report surveys were completed by consenting Master of Teaching pre-service teachers. The survey has demonstrated high levels of reliability (Lander et al., 2019) and assessed Master of Teaching pre-service teachers’ (i) willingness to integrate active teaching into professional practice (i.e. teaching placements); (ii) perceived impact of increased physical activity and breaking up sitting time on student outcomes; (iii) confidence to integrate specific active teaching strategies in the classroom; (iv) confidence to integrate specific active teaching strategies beyond the classroom (e.g. during recess); (v) competence to effectively integrate specific active teaching strategies across the school day; and (vi) perceived barriers to the delivery of active lessons. There were five to 15 items per construct and survey responses were based on a 5-point Likert scale (1 = strongly disagree to 5 = strongly agree); potential score ranges are provided in Table 3.

### Program implementation

Aspects of *implementation* (i.e. program fidelity and adherence) were collected via online lecturer adherence checklists. The checklists were completed by each lecturer delivering the TransformEd program at three time points across the 12-week feasibility trial (i.e. at weeks 3, 6 and 9). Lecturers indicated the implementation frequency on a 5-point Likert scale (1 = never, 2 = rarely, 3 = sometimes, 4 = often, 5 = as much as possible) under the five intervention components (see Table 2).

### Program feasibility

One online focus group discussion with senior academics (*n* = 2) and individual online interviews with lecturers who delivered TransformEd (*n* = 3) were conducted post-program by the lead researcher to investigate their perceptions of program feasibility and potential impact. Discussions ranged from 30 to 60 min, and semi-structured discussion prompts were used, guided by the eight areas of feasibility: demand, acceptability, implementation, practicality, adaption, integration, expansion and efficacy. ‘Member checking’ was performed during the focus group and interviews by summarising and relaying participant information to establish accuracy [45].

### Data management and statistical analysis

Descriptive statistics (i.e. median and interquartile ranges (IQRs) for summarised ordinal Likert scale variables, proportions for categorical variables) were used to compare Master of Teaching pre-service teachers’ willingness, feelings, confidence and competence, and perceived barriers total scores before and after the 12-week TransformEd program (only using participants who completed the survey at both time points). Descriptive statistics were also calculated to indicate lecturers’ self-reported *implementation* adherence to the program, which was summarised by each of the five key TransformEd components (i.e. lecturer implementation strategies, Table 2).

The senior academic focus group and lecturer interviews were transcribed verbatim and saved using a digital text editor (i.e. Microsoft Word 2018, Version 1806, Microsoft, Redmond, WA, USA). Guided by a qualitative descriptive approach, the transcripts were read, manually coded and collated into categories relevant to each of the eight areas of the feasibility framework. Coding reliability was conducted by two authors, who also completed reliability testing for approximately 35 min of the focus group data (~ 20%). Coding decisions were compared and divergent choices were discussed until agreement was found. The remaining data analysis was conducted by the lead researcher, and the accuracy of the coding process was verified.

## Results

In total, 21 master’s students completed the survey at both time points (55% response rate). The average age was 29 years and the majority were female (90.5%).

### Limited efficacy testing

Table 3 presents the program efficacy with regards to changes in pre-service teachers’ willingness to integrate active teaching into professional practice (teaching) placements; perceived impact of increased physical activity and breaking up sitting time on student outcomes; confidence to integrate specific active teaching strategies in the classroom; confidence to integrate specific active teaching strategies beyond the classroom (e.g. during recess); competence to effectively integrate specific active teaching strategies across the school day; and their perceived barriers, from baseline to post-program. Nearly all perception scores, including the summed total score, improved between baseline and follow-up, while willingness to integrate active pedagogies remained consistent.

**Table 3:** Difference between Masters pre-service teachers perceptions across time points for six components of physical activity integration in teaching (*n* = 21)

	T1 median (IQR)	T2 median (IQR)	Potential range
1. Willingness to integrate active pedagogies	25 (23; 27)	24.5 (23; 27)	6–30
2. Impact of active strategies on student outcomes	29 (28; 31)	31 (26; 34)	7–35
3. Confidence in class	61 (51; 67)	66 (59; 70)	15–75
4. Competence	16 (11; 20)	20 (19; 20)	5–25
5. Confidence out of class	12 (10; 15)	16 (13; 20)	5–25
6. Barriers	45 (36.5; 53)	40 (32; 53)	16–80
Total score (1 + 2 + 3 + 4 + 5–6)	95 (83; 110)	116 (92; 126)	42–174

*IQR* interquartile range

Sample size varies between *n* = 18–20 due to missing data

### Implementation

Three lecturers participated in the feasibility trial, two male and one female. Lecturer experience ranged from 5 years to over 20. Table 4 presents the lecturers’ adherence to the key implementation strategies across three time points. There was a noted increase in adherence from time point 1 to 2 in active lessons, active breaks and health lessons, which plateaued at time point 3. The total score increased consistently from time point 1 to time point 2 and time point 3.

**Table 4:** Mean scores of lecturer adherence over three time points for five components of TransformEd delivered by lecturers (*n* = 3)

	T1 median (IQR)	T2 median (IQR)	T3 median (IQR)	Potential range
1. Active lessons	15 (9; 16)	22 (20; 23)	23 (17; 23)	5–25
2. Active breaks	11 (7; 13)	20 (19; 23)	21 (18; 25)	5–25
3. Health lessons	11 (4; 17)	19 (12; 20)	19 (11; 20)	4–20
4. Active environments	8 (8; 8)	8 (4; 10)	11 (4; 13)	4–20
5. Active homework	11 (5; 13)	10 (5; 16)	10 (5; 17)	5–25
Total score (1 + 2 + 3 + 4 + 5)	56 (33; 67)	74 (69; 88)	77 (67; 93)	23–115

### Feasibility

#### Demand

All lecturers agreed that there was a need for a program that ‘*engaged their students* [*pre-service teachers*]* actively*’ in the course/unit content. A program that allowed pre-service teachers to ‘*experiment and try out*’ different pedagogies and approaches to teaching, and a resource that provided numerous activities and ideas for them to deliver the curriculum content in professional placements and their graduate year of teaching. Lecturers also shared that there was a need for a program that encouraged them [*lecturers*] to ‘*reflect on and refresh their own teaching*’.

This is a stimulus I guess for the lecturers to see how and where they use active pedagogies in their own teaching and supports them to continue to develop their own teaching practices around these active pedagogy. (Male lecturer)

#### Acceptability

The senior academics expressed that the content, alignment and pedagogical and instructional approaches of the program were valued and enhanced acceptability. It was shared that they ‘*needed something to engage the students [pre-service teachers] from the start and this type of approach to teaching was the solution*’.

All lecturers were satisfied with the professional learning and resources provided, and all participants perceived the program to be appropriate for the Master of Teaching degree. Lecturers reported that the design of the overall program and the structure of the resources enhanced the acceptability and appropriateness of the program.

… just how the course (TransformEd program) is set up in general, I think it was very easy to adopt. (Male lecturer)

In addition, the lecturers affirmed that the format of the program and the resources were well accepted by the master’s pre-service teachers.

The students (pre-service teachers) adapted to the program really quickly, they were engaged and seemed to really enjoy themselves. There was a real buzz, and it made the sessions fun and enjoyable. (Male lecturer)

Lecturers also unanimously suggested that the resources vastly improved pre-service teachers’ engagement in the unit content and agreed that it was appropriately aligned to unit learning objectives and ‘*very easy to integrate into the existing unit content and structure*’.

#### Implementation

All lecturers reported that they could, and did, implement the concepts and strategies successfully in their units. The lecturers expressed that their frequency and quality of implementation improved with practice and familiarity.

I found that the more I implemented the strategies the more I wanted to. There was an instant reward. You observed that the students [pre-service teachers] engaged and enjoyed the content more, so you were more inclined to include more of these resources into your lecturers. They just loved it. (Female lecturer)

Two lecturers shared that as the unit progressed the implementation ownership shifted from the lecturers to the pre-service teachers, who began to identify relevant active breaks or lessons and deliver them in lectures and seminars.

At the start we encouraged them to start implementing them [active breaks] themselves. Initially one or two of them would volunteer, but then they all wanted to. In the end it was more them than me, and they got a real buzz out of it. (Male lecturer)

#### Practicality

The senior academics agreed that the format, versatility, diversity and access to the program and resources enhanced practicality and use.

It’s not all or nothing, lecturers can find what they need and use it where they need. You don’t have to expect a complete overhaul, even small changes make a difference. (Male senior academic)

The lecturers unanimously agreed that the practicality of the program structure and the utility of the resources in the program were a big driver of implementation.

I was able to look at what I was going to be teaching each week in terms of the curriculum and identify the resource that linked very much to the activities and content I had already planned. (Female lecturer)

Further, lecturers expressed that the practicality and alignment of the program to their unit learning objectives made it ‘*easy to pick up and run with in any lecture*’. One lecturer shared that this is a practical and clever way to integrate physical activity more meaningfully into the curriculum.

It’s how we can and should integrate physical activity into the school day, not keeping learning and being physically active as completely separate constructs. So from that perspective it [TransformEd] was really easy because it has a lot of content and a lot of strategies that we can easily embed within the classroom. (Male lecturer)

#### Adaptation

Program adaptation was encouraged in the professional learning session, and the senior academics and lecturers shared that this knowledge reinforced ‘*the versatility and enhanced ownership of the program*’. One lecturer shared that the flexibility of the program enabled frequent adaptations and minor adjustments, which further enhanced effectiveness.

Because this wasn’t too prescriptive, not a one size fits all approach, we could make the adaptations we needed to really make it work for our unit. I had an intensive, and I was able to adapt the program to fit the more intense offering. (Male lecturer)

Another lecturer shared that the breadth of the program components and the diversity of the resources available (e.g. active academic lessons and active breaks) meant that different components could be focused on, and different resources could be used at varying points across the unit, depending on the content and focus of the lecture or seminar.

If I was focusing on classroom management, the active breaks were a great tool, whereas if I was looking at increasing engagement in English, the active lessons certainly increased engagement. It was just a great way to show how we can deliver the content in a fun way. (Female lecturer)

#### Integration

Lecturers unanimously shared that the concepts, strategies and pedagogies were effectively integrated in their units. They also reported that they felt as though they were organically integrating more of the program as the unit progressed. One lecturer expressed that this was largely driven by demand: ‘*the students [pre-service teachers] just loved it, so each week I had to find a way to add more in*’.

Further, one lecturer expressed that the pre-service teachers were integrating the strategies and pedagogies into their own teaching practices in subsequent professional placements, even though this was beyond the scope of the unit and program.

When you actually see it in practice, even though that wasn’t a defined outcome, but I think if we’re seeing it sort of infiltrating into their practices, it’s a pretty good sign because it’s clear that it’s really being adopted then. (Female lecturer)

Another lecturer expressed that a potential motivator for the master’s students to integrate the program was the clear evidence and rational behind it, and the fact that they had easy access to the evidence, as the publications were made available to them as part of the program. Providing clear evidence to demonstrate the positive and meaningful association between strategies the pre-service teachers were developing, and the health and education outcomes of the primary school student they teach, provided meaning and relevance to the program.

They’re really interested in the research behind this. They were sort of asking for more, you know, can you show me another research paper where these practices are being delivered and what are the effects? What are the different outcomes we are looking at? The more they understood the more they wanted to integrate it. (Male lecturer)

In addition, one lecturer shared that the breadth of the resources and the direct alignment with the mathematics curriculum meant that the program could be integrated across many of the concepts delivered in the unit.

I could link it into whatever topic I was doing at the time. So if I was doing multiplicative relations, I could bring in a multiplicative active break or active lesson task, and the same if we were doing fractions or decimals. There was pretty much a resource for everything we had to cover. (Male lecturer)

Lecturers shared some challenges to integration that were out of their control, but also expressed that they used these ‘real world challenges as teachable moments’. For example, one lecturer expressed that the room timetabled was not conducive to movement:

The room was an electrotype theatre room, which wasn’t ideal anyway for a tutorial, but we made it work and we used space in the corridors and things like that, so I used that as a teachable moment because I was able to explain to the students and say, look, we’re doing this activity, you know, you might have to move from one group to another and we’ve got limited space, but this is what it might be like in your classroom. So how can we adapt? How can we modify? How can we be a little bit more flexible? So that worked really well. (Female lecturer)

While another lecturer shared that the short length of the unit provided a challenge, the subsequent placement experience provided a great opportunity for the pre-service teachers to apply the concepts covered in lecturers and tutorials in their own teaching practice.

Probably the main challenge for this one was the short time span that we had to deliver the program because it was a condensed delivery. We only had the students [pre-service teachers] for six weeks. But they had a four-week placement block at the end, and they did utilise a lot of the strategies, they sort of embedded them in their own classroom, just like they experienced in the tutorials that we had. (Male lecturer)

#### Expansion

Regarding program expansion, senior academics and lecturers shared that there was a need for the program to be expanded across more units in the Master of Teaching degree.

The students got it in three courses [units] this year, but for it to really work, they need to see it in every unit they receive. It needs to be reinforced over and over, so they just see it as common practice. (Male lecturer)

I think it’s consistency across multiple different courses [units] throughout the student’s [pre-service teachers] whole program. If they’re getting it only in one or two courses [units], they might be getting different messages. But I think to make it more sustainable and for students to adopt it more readily, it needs to be embedded across the whole program. (Male lecturer)

Indeed, many shared that to have a lasting impact on the pre-service teachers, expansion was pivotal.

This approach is something that could easily apply to all lessons all units and all years. It could and should be part of what they’re doing anyway, it’s not separate, it’s such an important way to learn to teach. Without the students even knowing that this is a research program, you know, it’s become part and parcel of the lesson automatically. This needs to happen in all units. (Male lecturer)

In addition, lecturers perceived that the program could be expanded beyond the Master of Teaching degree quite easily.

I don’t think it’ll be hard for you to get it incorporated into the classrooms. To be honest I think that if you do what you’re doing now in terms of the master’s students, where they have embraced it so well, I think you could get that into the School of Education as well in terms of the four-year course, and just make that part of it, like I have with the numeracy. (Female lecturer)

All lecturers agreed that the TransformEd program should be expanded to become a formalised component of the professional practice experience.

Our students [pre-service teachers] often learn more about their own teaching from placement than from the lecturers and tutorials. Without it being a formalised part of their course the students [pre-service teachers] were embedding the strategies in their practice, and immediately seeing the impact with the children. This highlighted the relevance, the need and the impact. We should harness this and make it a formalised part of their lesson plans, lesson reflections and assessments. (Female lecturer)

#### Efficacy

Senior academics and lecturers reported that the program was ‘*effective on a number of levels*’. One lecturer expressed that it had an ‘*immediate and positive impact on the classroom environment*’ in the lectures and tutorials. As such, the program had ‘*an added benefit of enhancing the health outcomes of the preservice teachers*’.

Because it’s something where you can see the direct effect on students [pre-service teachers] in terms of engagement and enjoyment in those lessons. (Male lecturer)

Another expressed that training the future teachers was an effective way to change teaching practice.

I think we need to change the attitude of the teachers when we’re training them to be able to adapt more, not get locked into one way of teaching. I think maybe through the universities and the pre-service courses using a program like this you might be able to change the younger teachers and gradually change practice. (Male lecturer)

Another suggested that the effectiveness went ‘*beyond just increasing activity*’ and explained that it reinforced or consolidated the core content of the lessons, as such the program improved the learning outcomes.

It’s not just getting them up out of their seat, it’s more than that, it’s a real learning experience, you know, getting them to understand the content just through that method of being a bit more active than usual. (Male lecturer)

Another lecturer suggested that the clear connection between how the pre-service teachers were being taught in the university setting and how they needed to teach during placements in schools was a real ‘*value add*’.

The relevance and alignment meant that they could see the link directly through how they could implement it into school. (Male lecturer)

The senior academics and lecturers unanimously expressed that the true impact of the program was demonstrated during pre-service teacher placements, where the concepts, strategies and pedagogies experienced during lecturers and tutorials were planned and delivered in their own teaching practice.

The joy for me was when they do their placement course, and I could actually see that they’d integrated some of the activities into their lessons and were seeing the rewards. (Female lecturer)

I think the students were really receptive to what we were doing, and I could see that in their own teaching. Both in their peer teacher practices and assessments they had to do for the course. Then in the four-week placement block at the end they utilised a lot of the strategies they were embedded in. (Male lecturer)

Concrete evidence [of effectiveness] when they submitted their portfolio that they’ve got these little active breaks in there and that’s a real highlight for me to be able to see if they were being taught it and then they were actually implementing it in their own practice. (Female lecturer)

#### Progression criteria for TransformEd based on CONSORT-informed feasibility domains

The assessment of *TransformEd* against CONSORT-informed feasibility progression criteria provides a foundation for progressing to a subsequent controlled trial to rigorously evaluate the intervention’s efficacy [43]. As summarised in Table 5, the program demonstrated strong acceptability among pre-service teachers and lecturers, supported by high levels of implementation fidelity and evidence of increasing uptake over time. Preliminary efficacy findings suggest positive improvements in teaching confidence and competence, while recruitment and data collection procedures were both effective and manageable within the postgraduate setting. In addition, strong stakeholder engagement and alignment with institutional priorities highlight the potential for scalability and sustainability of the intervention.

**Table 5:** Progression criteria for TransformEd based on CONSORT-informed feasibility domains

**Feasibility Domain**	**Definition (CONSORT-based) ** [43]	**Evidence from TransformEd Feasibility Study**	**Assessment**
Demand	Interest or need for the intervention	Lecturers and senior academics expressed a clear need for a program that actively engages pre-service teachers and provides tools for use in professional placements	**Met**
Acceptability	Suitability and satisfaction with the intervention	Lecturers and students found the program engaging, aligned with unit objectives and easy to adopt; strong satisfaction with resources and structure	**Met**
Implementation	Fidelity and quality of delivery	Increasing lecturer adherence across time points; qualitative data confirmed high implementation success and uptake by both lecturers and students	**Met**
Practicality	Ease of use and fit within existing systems	Program was viewed as flexible, adaptable, and easy to embed within lectures; resources matched existing content and learning objectives	**Met**
Adaptation	Flexibility to modify components	Lecturers successfully adapted content for different delivery formats (e.g. intensive vs. standard); modifications improved contextual fit and effectiveness	**Met**
Integration	Incorporation into routine practice	Strong lecturer and student integration of active strategies into lessons; pre-service teachers applied strategies in placements beyond study scope	**Met**
Expansion	Potential for scale-up or broader application	Stakeholders supported expansion across more units and programs; potential noted for integration into undergraduate and other professional education streams	**Met**
Preliminary efficacy	Early indications of intended effects	Improvements observed in confidence, competence, and perceived student impact; qualitative data confirmed perceived effectiveness and practical application during placements	**Met (preliminary and exploratory)**

## Discussion

This study contributes to accumulating evidence for the utility and effectiveness of embedding the TransformEd program in initial teacher education [34, 36, 37]. The interview and focus groups results showed that the TransformEd program was positively received by Master of Teaching pre-service teachers, resulting in several improvements in survey-measured confidence and competence in regard to strategies that increase school children’s health, wellbeing and education outcomes. Strategies targeting physically active lessons, active breaks and health content were well adopted by lecturers and implementation increased over time. The lecturers and senior academics had positive perceptions of the program, highlighting its feasibility in postgraduate initial teacher education.

Overall, the program resulted in several meaningful and promising changes in pre-service teachers perceived competence and confidence to integrate pedagogical strategies targeting physical activity in and beyond the classroom. This is a promising finding considering that teacher-related factors (e.g. low confidence, knowledge and motivation) have been cited as barriers to physical activity implementation in primary schools [46]. Without sufficient training, teachers may lack the knowledge and confidence to embed effective strategies for promoting physical activity among their students. Importantly, in whole-of-school approaches to physical activity that expand the reach of physical activity programs beyond physical education and school sport [47]. Although quality physical education is the cornerstone of comprehensive school physical activity programs, primary school teachers face institutional barriers (e.g. overcrowded curriculum and low subject priority) that hinder their ability to deliver effective physical education [46, 48]. As such, TransformEd provides an approach that may mitigate these institutional barriers, instead providing pre-service teachers with innovative strategies to increase physical activity outside of physical education. In a previous feasibility study of TransformEd targeting undergraduate teacher education, school principals and academics perceived that physically active teaching across core subjects (e.g. mathematics, English, science) could improve both the health and educational outcomes for children and should become part of every future teacher’s ‘tool kit’ [34]. Thus, as TransformEd positions physical activity as a teaching tool and a vehicle for increased student engagement, the program may simultaneously increase children’s physical activity, health and improve their learning.

In the present study, efficacy of TransformEd was viewed positively by Master of Teaching pre-service teachers, with the largest effect observed for pre-service teachers’ confidence to deliver physically active pedagogy in the classroom. A similar effect was observed in an earlier feasibility study of the TransformEd program in undergraduate pre-service teachers [34]. While significant improvements were observed in both studies, participants in the current study reported a much higher baseline value (57.9 compared with 20.1). This difference may be due to characteristics of the study sample; for example, Master of Teaching pre-service teachers with a mean age of 29 years in the present study compared with undergraduate students mostly aged 17–21 years in the previous study. In a recent TransformEd implementation trial [37], small improvements in teacher confidence were not statistically different from the control group. In sum, the current study provides preliminary evidence that TransformEd may be effective in improving Master of Teaching pre-service teachers’ confidence in delivering physically active strategies, including for those teachers that have already high levels of confidence; however, these results should be confirmed in a larger, more comprehensive trial.

Lecturers reported mixed results regarding implementation. Adherence to all five components increased over time (although negligible improvement was found for ‘active environments’ and ‘active homework’), with the largest improvements in physically active learning and active break strategies. Physically active learning and classroom activity breaks have been shown to benefit a range of physical activity, health and educational outcomes in children [49–51]. However, there is debate regarding the optimal intensity of physical activity for achieving the best outcomes [51]. For example, high-intensity physical activity has been shown to elicit significant improvements in a range of cognitive and psychological health outcomes [10, 52]. The practical implementation of high-intensity physical activity is often operationalised in the form of short classroom activity breaks to maximise time efficiency [53]. Regardless of intensity, physically active learning strategies that provide a direct link to curriculum and learning outcomes (i.e. active academic lessons and active breaks) may be more readily adopted into subjects that have an academic focus (e.g. literacy or numeracy units). This may explain why ‘active environment’ strategies were less adhered to here and previously [36, 37], if they had lower perceived alignment with core curriculum.

The combined provision of resources and strategies to implement concepts and pedagogies into lecturers’ own teaching appeared to be impactful. Lecturers perceived the resources to be highly effective for their own teaching, but also mentioned how they were readily accepted and adopted by pre-service teachers. Indeed, the combined impact of provision of resources, seeing them modelled in action by the lecturer, and practising them in peer-teaching scenarios, may have been a driving factor in improving pre-service teachers’ teaching confidence and competence. Previous research has highlighted that the provision of resources and materials as part of professional development is highly valued by in-service teachers, particularly when their content knowledge is limited [54]. The TransformEd program and associated resources present an innovative teaching and learning model that positions physical activity as a teaching tool and part of an instructional model [42]. The fusing of physical activity research in education practice may be an effective and sustainable way to develop pre-service teachers’ confidence and competence in the delivery of active pedagogies, and consequently improve primary school students’ learning, physical activity and health outcomes.

The flexibility and versatility offered by TransformEd enabled lecturers to adopt the program to suit their unit and enhance program ownership. Specifically, lecturers spoke about how they were able to look at the program resources and strategies and apply these to their pre-existing course material and planned learning. Indeed, this versatility indicates that the TransformEd resources and strategies were adaptable to different teaching styles, subject areas and contexts, adding value for those delivering the program. This concept has also been raised in previous evaluations of the TransformEd program across different university settings [36, 37]. Therefore, adaptation and flexibility appear to be key drivers for implementation of the TransformEd program. Although the focus of this particular study was to determine feasibility, program adaption and flexibility are key considerations for future definitive trials and scale-up interventions [55].

Lecturers and senior academics consistently put forward the potential impact of the TransformEd program in initial teacher education. One of the main themes was the need for program expansion to more units and years of the degree, which is consistent with results from previous feasibility and implementation work [34, 37]. This approach would help reinforce key concepts and strategies, and more closely align with the overarching aims of effective teacher professional development. For example, ‘duration’ and repeat exposure is an important feature of effective professional development, yielding greater results than ‘one-off’ external workshops [33]. Further, integrating innovative behavioural, pedagogical and environmental strategies into initial teacher education rather than, or in addition to, professional development may be a more effective and sustainable approach. Our findings in postgraduate initial teacher education contribute to accumulating evidence supporting the potential impact of TransformEd in initial teacher education [34, 36, 37]. The findings also support that impact of TransformEd may be enhanced if pre-service teachers engage with the program more frequently and receive greater exposure throughout the duration of their degree.

One of the key themes from the focus group discussion and interviews was the relevance of TransformEd in initial teacher education. TransformEd was seen to be a feasible approach to help bridge the gap between theory and practice, which is a challenge in initial teacher education [56]. Of note, the theory–practice divide was a prevalent theme in recent TransformEd implementation trials [36, 37], in which lecturers acknowledged that there was ‘real demand’ for programs that strengthen the connection between pre-service teacher education and teaching expectations in schools [37]. A nearly identical sentiment was raised in the current study, with one lecturer highlighting the importance of preparing pre-service teachers for their professional placements by aligning university teaching with real-world expectations. By allowing pre-service teachers to experience key concepts and strategies firsthand as learners before assuming the role of teacher, TransformEd appears to successfully address this issue. This integration of theory and practice aligns well with proposed enhancements to initial teacher education in Australia [57], and it is therefore promising to see that TransformEd is perceived to support this crucial link.

One novel finding that emerged from the qualitative data was lecturers’ perceived impact of TransformEd on pre-service teachers’ enjoyment and engagement. This finding adds valuable insight into the potential effectiveness of TransformEd, and likely aided program implementation. The observed positive effects on the classroom environment, reported by one lecturer, highlight the program’s immediate benefits. While this study did not directly investigate the effects on pre-service teachers’ classroom engagement, the consistency of these findings with previous experimental research underscores the significance of incorporating classroom activity breaks and physically active learning in all educational settings [22, 58]. Lynch and colleagues’ recent systematic review further supports the feasibility and efficacy of such interventions in university settings [59]. Future studies exploring the direct effects of TransformEd on pre-service teachers’ classroom engagement, in addition to health and wellbeing outcomes, could provide additional evidence for the program’s broader educational impact.

TransformEd demonstrated strong feasibility, with high acceptability, fidelity and uptake, alongside promising improvements in teaching confidence and competence. Recruitment and data collection processes were effective within the postgraduate setting, and strong institutional alignment supports the program’s scalability and sustainability, providing a clear rationale for a subsequent controlled trial [43]. Moving forward, next steps should include conducting a controlled trial to test the effectiveness of the program, expanding to multiple institutions to assess generalisability, increasing the sample size to ensure adequate statistical power, refining implementation supports for lecturers and exploring the long-term impact of the program on school children's health, wellbeing, and learning outcomes [43].

### Strengths and limitations

There are several strengths of our study that offer a unique contribution to the field. First, to the best of our knowledge, this is the first study to examine the feasibility of embedding physically active pedagogy into a postgraduate initial teacher education program. Second, the mixed methods study design provides comprehensive insight into the feasibility as perceived by relevant stakeholders including pre-service teachers, lecturers and senior academics. Third, TransformEd was delivered across several courses or units in the Master of Teaching program rather than a single course or unit.

Several limitations should also be acknowledged. First, small sample sizes and recruitment from only one university limit the overall generalisability of the findings. Second, due to the nature of this feasibility study, a control group was not included, and as such, causal inferences cannot be made. Therefore, these preliminary efficacy findings should be interpreted with caution and confirmed in a larger controlled trial. Finally, the collection of qualitative data may have been subject to social desirability bias due to the lead researcher’s involvement in the interviews and focus groups. In future, having an independent researcher conduct interviews may help mitigate this issue.

## Conclusion

This study provides support for the feasibility and preliminary efficacy of the TransformEd program in the context of postgraduate initial teacher education. TransformEd resulted in several changes in pre-service teachers’ competence and confidence to deliver physically active pedagogy that increase school children’s health, wellbeing and education outcomes. Lecturers delivered the program with moderate levels of implementation and adhered most to physically active learning and active breaks. It was universally agreed that TransformEd represents a feasible, acceptable and potentially impactful program; however, future research with larger, more representative samples is needed.

## Supplementary Information

Additional file 1.

## Data Availability

The datasets during and/or analysed during the current study available from the corresponding author on reasonable request.
